# A Single-Step Protein Extraction for Lung Extracellular Matrix Proteomics Enabled by the Photocleavable Surfactant Azo and timsTOF Pro

**DOI:** 10.1016/j.mcpro.2025.100950

**Published:** 2025-03-17

**Authors:** Anna G. Towler, Andrew J. Perciaccante, Timothy J. Aballo, Yanlong Zhu, Fei Wang, Sarah Lloyd, Kuniko Kadoya, Yupeng He, Yu Tian, Ying Ge

**Affiliations:** 1 Department of Chemistry, University of Wisconsin-Madison, Madison, Wisconsin, USA; 2 Molecular and Cellular Pharmacology Training Program, University of Wisconsin-Madison, Madison, Wisconsin, USA; 3 Human Proteomics Program, School of Medicine and Public Health, University of Wisconsin-Madison, Madison, Wisconsin, USA; 4 Quantitative Translational & ADME Science, AbbVie Bioresearch Center, Worcester, Massachusetts, USA; 5 Discovery Immunology, Pharmacology and Pathology, AbbVie, Inc, North Chicago, Illinois, USA; 6 Allergan Aesthetics, an AbbVie Company, Irvine, California, USA

**Keywords:** extracellular matrix, matrisome, lung, proteomics, photo-cleavable surfactant, mass spectrometry, tissue

## Abstract

The extracellular matrix (ECM) is a dynamic, complex network of proteins, collectively known as the “matrisome,” which not only provides essential structural support to cells and tissues but also regulates critical cellular processes. Dysregulation of ECM is implicated in many diseases, underscoring the need to characterize the matrisome to better understand disease mechanisms. We have previously developed a dual-step protocol enabled by the photocleavable surfactant Azo for the extraction of ECM proteins from tissue using pH-neutral decellularization followed by solubilization by Azo. While effective for characterization of ECM proteins, such a dual-step protocol requires two extracts per sample, limiting the throughput and complicating the comparison of protein quantitation across different extraction conditions. Here, we develop a single-step Azo-enabled protein extraction for the solubilization of ECM proteins from lung tissue to improve the throughput for studies with large sample sizes. Using this method, we identified 324 ECM proteins, including 137 core ECM and 187 ECM-associated proteins. Core ECM proteins including elastin, fibronectin, and fibrillar collagens were reproducibly identified and quantified. We observed a 94.6% overlap in the ECM proteins identified between the single-step and the dual-step Azo extracts, indicating the single-step Azo extraction achieves ECM protein coverage comparable to the dual-step extraction. Overall, we have demonstrated that this single-step Azo extraction is not only highly efficient but also comprehensive for ECM protein identification and quantification, making it a powerful method for ECM proteomics, especially for studies with large sample sizes.

## Highlights

• We developed a single-step Azo-enabled extraction method for efficient ECM proteomics.
• More than 300 ECM proteins from human lung tissues, including core ECM and ECM-associated proteins, were identified.
• The single-step Azo extraction achieved comparable ECM protein coverage to the dual-step extraction method.
• Key ECM components, such as elastin, fibronectin, and fibrillar collagens, were identified.
• This method greatly improves throughput and reproducibility, enabling large-scale ECM proteomics studies.

## Graphical Abstract

**Figure:**
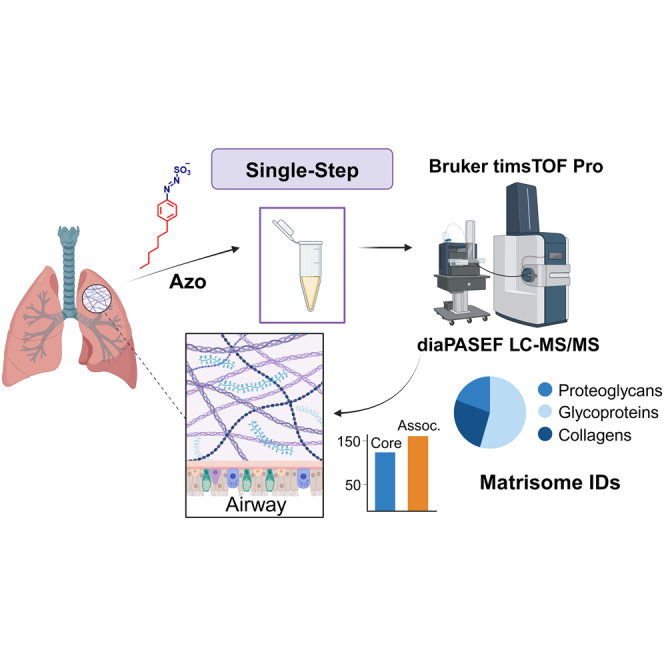

## In Brief

We developed a single-step extraction method for extracellular matrix (ECM) proteomics using the photocleavable surfactant Azo, significantly improving throughput and reproducibility. This approach identified over 300 ECM proteins from human lung tissues, including both core ECM and ECM-associated proteins, with proteome coverage comparable to the dual-step extraction. This method streamlines ECM proteomics, enabling comprehensive ECM protein identification and quantification for large-scale studies to understand the role of ECM in health and disease.

The extracellular matrix (ECM) is a dynamic network of proteins that play a critical role in the organization of cells and tissues (1, 2, 3). The term “matrisome” was coined to describe the set of genes which encode for the ECM and includes core matrisome proteins consisting of glycoproteins, proteoglycans, and collagens as well as matrisome-associated proteins including secreted factors, ECM-affiliated proteins, and ECM regulators (4, 5). ECM proteins provide physical scaffolding to cells and facilitate cellular processes including migration, communication, proliferation, and differentiation through biochemical signals (1, 3, 6, 7). The ECM can be structurally divided into the basement membrane and the interstitial matrix. The basement membrane is located where the epithelium meets connective tissue and is composed of non-fibrillar type IV collagen, structural glycoproteins including laminins, and the heparan sulfate binding protein perlecan (3, 8). The interstitial matrix is present alongside the basement membrane as well as between connective tissues and is composed of fibrillar collagens, fibronectin, and elastin (3, 8). Under normal physiological conditions, the ECM undergoes structural remodeling, but dysregulation of ECM homeostasis can lead to tissue stiffening and are characteristic of many diseases (3).

In the lungs, the ECM includes both basement membrane and interstitial matrix components (9, 10). The pulmonary ECM is responsible for the elastic structure of the lungs and is integral for cellular communication and injury repair (9, 11). ECM remodeling is critical for the maintenance of the pulmonary ECM, and the ECM undergoes extensive changes in composition throughout lung development (10, 11). However, abnormal ECM deposition and tissue remodeling drive the progression of lung diseases, including fibrosis (11, 12, 13, 14). Lung stiffness resulting from ECM dysregulation is intricately tied to the pathogenesis of many diseases including chronic obstructive pulmonary disease (COPD), lung cancers, and pulmonary fibrosis (6, 9, 15, 16). As such, there is an urgent need to characterize the role of the ECM in lung health and disease (11, 10,14).

Mass spectrometry (MS)-based proteomics is a powerful analytical tool for the identification and relative quantification of the ECM proteome (6, 17, 18). We previously developed a dual-step ECM proteomics workflow enabled by the photocleavable surfactant Azo (19). More recently, we report a strategy for ECM proteomics enabled by Azo and data-independent acquisition parallel accumulation serial fragmentation (diaPASEF) (20) for the analysis of matrisome proteins from human lung tissue (21). The protein extraction protocol for these methods involves decellularization followed by a second extraction with Azo (22, 23), resulting in the analysis of two extracts from a single tissue sample (19, 21). Although the dual-step extraction is reproducible and efficient for ECM proteomics experiments with a small number of samples, it poses limitations for studies with large cohorts by necessitating the analysis of two extracts per biological sample (19, 21). A dual-step extraction increases the time needed for sample preparation, data acquisition, and analysis, and this is particularly cumbersome in human studies which must account for variability from genetic, environmental, and lifestyle factors with large sample cohorts (24, 25).

Herein, we have developed a single-step Azo-enabled extraction for matrisome proteins from lung tissue, reducing the number of samples to be analyzed by half compared to the dual-step extraction and significantly improving the throughput for large biological cohorts. Our results show that this single-step Azo extraction achieves comparable matrisome coverage to that of our dual-step extraction, underscoring its robustness and efficiency for high-throughput applications in large-scale studies.

## Experimental procedures

### Experimental Design and Statistical Rationale

In this study, we used three extraction replicates (n = 3) for both single-step and dual-step extractions. LC-MS/MS analysis was performed on all extracts, and lung tissue matrisome protein coverage was compared across extractions. DIA data were searched against an *in silico* spectral library, and protein identifications were matched to the Naba Matrisome Database (4, 5) to determine total ECM protein identifications across extracts. Overlap in protein identifications and strong Pearson correlation coefficients across extraction replicates demonstrated single-step and dual-step extractions are highly reproducible. The overlap in matrisome protein identifications between extract types demonstrated the degree of similarity in ECM protein coverage between single-step and dual-step extraction methods. Total protein intensity resulting from matrisome proteins and peptide numbers were compared between extracts to assess differences in matrisome coverage across single-step and dual-step methods. Student’s t-tests were performed to determine significance in the numbers of peptides detected between single-step and dual-step Azo extracts.

### Chemicals and Reagents

All chemicals and reagents were purchased from Millipore Sigma and Thermo Fisher Scientific unless otherwise stated.

### Lung Tissue Collection

Human lung tissue was collected in collaboration with the University of Wisconsin Organ and Tissue Donation Service. Deidentified donor tissue was assigned a numerical code and information regarding only the donor’s sex, age, and relevant pulmonary history was recorded. Lung tissue collection was approved by the Institutional Review Board of the University of Wisconsin–Madison (Study # 2011–0868). Each lobe of the lungs was dissected and tissue collected from defined regions upon receipt at 4 °C followed immediately by snap freezing in liquid nitrogen (21). Lung tissue was stored at −80 °C. The human studies reported here abide by the Declaration of Helsinki principles.

### Lung Tissue Protein Extraction

For the dual-step extraction, we used the protocol for the extraction of ECM proteins as reported previously (21). For the single-step extraction, we followed the Azo extraction step previously described in our dual-step protocol (21). To summarize, 200 mg of lung tissue from the left central lower lobe of the lungs was cut and cryopulverized in liquid nitrogen (Thermo Fisher Scientific, Cellcrusher Kit). For dual-step and single-step samples, 30 mg cryopulverized tissue was weighed out in a cold room at 4 °C. Two rounds of washing with Dulbecco’s phosphate-buffered saline with 1× Halt Protease Inhibitor Cocktail (Thermo Fisher Scientific) were carried out to deplete abundant blood proteins (21).

For the single-step Azo extraction, 100 μl Azo extraction buffer (0.5% Azo, 1× Halt, 1 mM Na_3_VO_4_, 1 mM PMSF, 2.5 mM EDTA, and 1 mM DTT) was added to each sample and manually homogenized, briefly. Samples were then probe sonicated at 20% amplitude for three cycles at 3 s each, heated to 95 °C on a shaker at 300 rpm for 30 min, and sonicated in a water bath for 60 min at room temperature. Finally, samples were spun down in the centrifuge for 10 min at top speed and the supernatant was collected and labeled ‘Azo’ (21).

The dual-step protocol involved solubilization of the cryopulverized and DPBS-rinsed tissue in 100 μl decellularization buffer (40 mM HEPES (pH = 7.9), 60 mM NaF, 1× Halt, 1 mM Na_3_VO_4_, 1 mM PMSF, 1 mM DTT, and 25 mM EDTA). The samples were manually homogenized and centrifuged for 15 min at top speed. Decellularization with 100 μl buffer was performed for three rounds, and the supernatants were pooled together and labeled ‘Decell’. The Decell extracts underwent five rounds of buffer exchange in 25 mM ammonium bicarbonate using 10 kDa molecular weight cut-off filters (21). The remaining pellet was extracted according to the protocol described for the single-step Azo extraction.

Total protein concentration was determined using a Pierce Bradford Plus Protein Assay Kit (Thermo Fisher Scientific). The samples were normalized in 0.1% Azo in 25 mM ABC for bottom-up sample preparation. The samples were reduced in 25 mM TCEP (pH = 8), alkylated with 30 mM chloroacetamide, and enzymatically digested with Trypsin Gold (Promega) overnight at 37 °C. Formic acid was used to quench digestion, and UV irradiation was performed using a 6 W UV lamp for 15 min. Peptide desalting was completed with Pierce C18 Pipette Tips (Thermo Fisher Scientific). Final peptide concentrations were determined by NanoDrop One using the A205 program (Thermo Fisher Scientific), and the samples were transferred to LC-MS vials for analysis (21).

### Bottom-Up Proteomics Data Acquisition

Bottom-up data acquisition used similar parameters as previously established (21). Briefly, peptides were analyzed by LC-MS/MS on a nanoElute (Bruker Daltonics) nanoflow ultra-high pressure LC system (UHPLC) coupled to a timsTOF Pro (Bruker Daltonics) operating in data-independent acquisition (DIA) mode with parallel-accumulation serial fragmentation (PASEF) (diaPASEF) (21). Approximately 200 ng peptides were injected into an Aurora Ultimate C18 column (25 cm × 75 μm inner diameter x 1.7 μm particle size) (IonOpticks). The diaPASEF parameters were as follows: the inverse reduced mobility (1/K_0_) scan range was from 0.6 to 1.43, the mass scan range was 400 to 1200 m/z, there were 32 isolation windows with a width of 26 m/z and a 1 m/z overlap between cycles, 1/K_0_ spanned 0.3 V×s/cm^2^ for each isolation window, and the cycle time was 1.8 s. Collision energies were assigned as a function of 1/K_0_, beginning with 20 eV at 1/K_0_ = 0.6 and cycling through 60 eV at 1/K_0_ = 1.6. Detailed diaPASEF parameters are recorded in Supplementary File 1. The total ion current (TIC) intensity of the samples was compared to the TIC intensity of 200 ng peptide injections of K562 whole cell lysate standard (Promega) to verify the peptide injection amount. The LC method used the same gradient previously reported with our dual-step extraction (21). The MS method used was also consistent with the method reported in the previous dual-step method, with a mass-to-charge (m/z) range set between 100 m/z and 1700 m/z (21).

### Bottom-Up Proteomics Data Analysis

Bottom-up data analysis was performed similarly as we reported previously (21). Briefly, raw MS/MS data was searched in DIA-NN v1.8.1 (26, 27) against a spectral library generated using a human FASTA from UniProt database UP000005640 consisting of approximately 20,000 entries (canonical, accessed 08 April 2024).Trypsin/P was the selected protease, and the maximum number of missed cleavages was set to 2. Fixed modifications included N-terminal methionine excision and carbamidomethylation, and variable modifications included methionine oxidation and N-terminal acetylation. MS1 accuracy was 10 ppm, MS2 accuracy was 20 ppm, and the precursor false discovery rate was set to 1% (21). Label-free quantification was performed in DIA-NN using MaxLFQ (26, 28). Proteins were filtered out if not present in two-thirds of biological replicates, and missing values were imputed using least squares imputation. The Decell extracts were searched separately from the dual-step and single-step Azo extracts. Comparison between single-step and dual-step protein identifications was accomplished with DeepVenn (29) and R. Matrisome protein identifications were determined based on gene matches to the Naba Matrisome Database (4, 5). Pearson correlation plots were generated using Perseus (30). Gene ontology-cellular component analysis (GOCC) was completed with PANTHER (31), and STRING protein-protein (32) analysis was performed on the matrisome proteins identified in the single-step Azo extracts.

## Results and Discussion

### A Single-Step Extraction of Extracellular Matrix Proteins from Lung Tissue

**Fig. 1:**
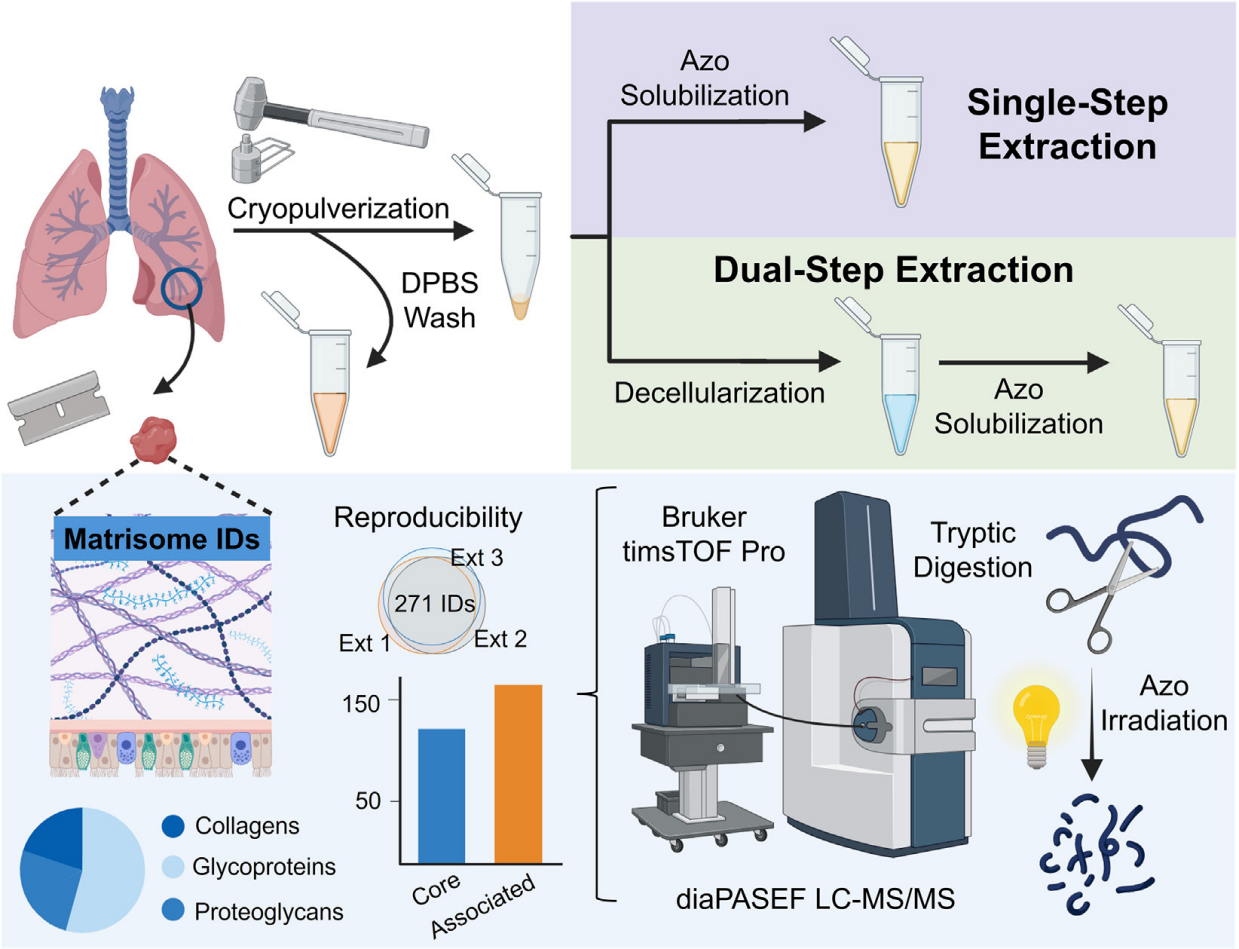
**Schematic on single-step *versus* dual-step extraction of extracellular matrix proteins from lung tissue followed by bottom-up proteomics analysis.** Lung tissue was cryopulverized and rinsed to deplete abundant serum and blood proteins. For the dual-step protocol, tissue was decellularized in a HEPES buffer followed by extraction of the insoluble pellet with Azo (21). For the single-step protocol, tissue was solubilized in an Azo-based buffer for the extraction of extracellular matrix proteins without a prior decellularization step. The dual-step decellularized and Azo extracts and the single-step Azo extracts were enzymatically digested in trypsin followed by Azo degradation. Approximately 200 ng peptides were injected and analyzed by LC-MS/MS for each replicate. Parts of this figure were generated using BioRender.

Our single-step protein extraction protocol improves throughput for ECM proteomics by reducing the number of extracts for analysis by half (Fig. 1). It reduces the time required for protein extraction by eliminating the decellularization step which requires multiple rounds of buffer exchange with molecular weight cutoff filters prior to enzymatic digestion (21). Additionally, substantial time is saved on desalting peptides following trypsin digestion with only a single extract to analyze for each biological sample. Data acquisition time is reduced by half, and data processing is simplified to analyze a data set with protein identifications from a single extraction type which eliminates the challenges associated with comparing protein identifications and quantification across multiple extractions.

### Reproducibility Across Dual-Step and Single-Step Extraction Replicates

**Fig. 2:**
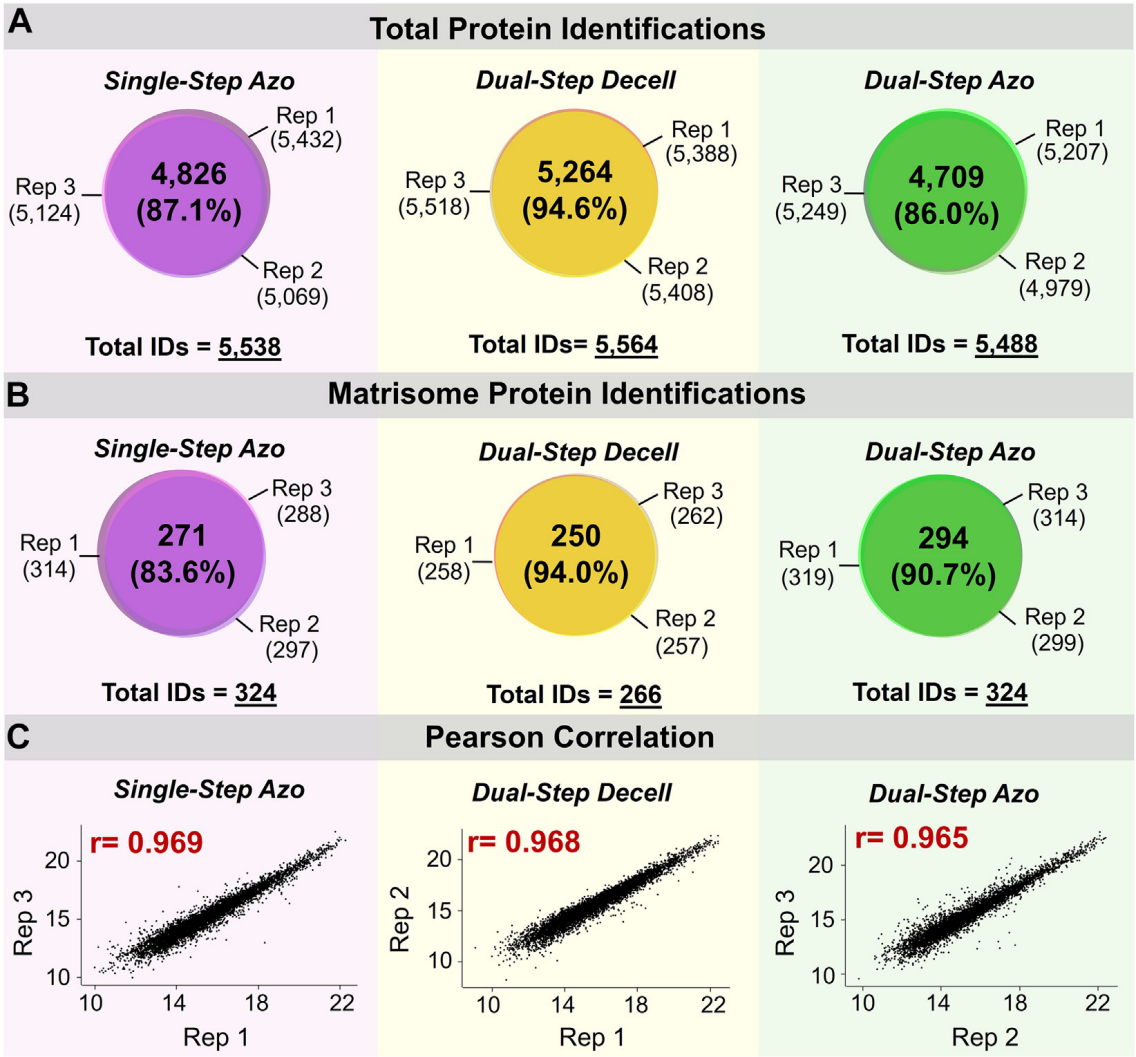
**Both single-step and dual-step extraction methods are reproducible.***A*, Venn diagrams demonstrate total protein identifications in the single-step Azo extracts, dual-step Decell extracts, and dual-step Azo extracts. *B*, Venn diagrams demonstrate matrisome protein identifications in the single-step Azo extracts, dual-step Decell extracts, and dual-step Azo extracts. *C*, Representative Pearson correlation plots for single-step Azo, dual-step Decell, and dual-step Azo extraction replicates.

Both single-step and dual-step Azo-based extraction methods demonstrate reproducibility across extraction replicates. Across n = 3 single-step Azo extracts, between 5069 and 5432 total proteins were identified with an 87.1% overlap in protein identifications. In total, 5538 unique proteins were identified in single-step Azo extracts. In the dual-step Decell extracts, we identified between 5388 and 5518 proteins with 94.6% overlap in identifications across extraction replicates, and in total, 5564 unique proteins were detected. In the dual-step Azo extracts, protein identifications ranged from 4979 to 5249 with an 86.0% overlap in identifications across extraction replicates, and in total, 5488 proteins were quantified (Fig. 2*A*). Total protein identifications were searched against the Naba Matrisome Database (4, 5) to determine the number of matrisome proteins quantified in single-step Azo, dual-step Decell, and dual-step Azo extracts. In single-step Azo extracts, the number of matrisome proteins detected in each replicate ranged from 288 to 314 with an 83.6% overlap in matrisome identifications. A total of 324 unique matrisome proteins were identified in n = 3 single-step Azo extracts. Matrisome protein identifications in the dual-step Decell extracts ranged from 257 to 262 proteins with a 94.0% overlap in identifications across n = 3 extraction replicates and a total of 266 unique matrisome proteins. In the dual-step Azo extracts, the number of matrisome proteins quantified was between 299 to 319 with a 90.7% overlap in identifications across n = 3 extraction replicates, resulting in 324 unique matrisome proteins (Fig. 2*B*). Consistency in the number and identification of proteins detected across single-step and dual-step extraction replicates demonstrates the reproducibility of both extraction methods.

Total ion chromatograms are overlaid for single-step Azo, dual-step Decell, and dual-step Azo extraction replicates to show reproducibility in spectral intensity (supplemental Fig. S1*A*). For single-step Azo extracts, an average of 31,479 peptides were identified in each extraction replicate with 19,988 peptides detected in common between replicates. An average of 35,467 peptides were identified in dual-step Decell extracts and 35,717 in dual-step Azo extracts (supplemental Fig. S1*B*). Log2-transformed total peptide intensities follow a normal distribution for both single-step and dual-step extracts (supplemental Fig. S1*C*). Pearson correlation plots for the extraction replicates corresponding to the median Pearson correlation coefficient emphasize a strong positive correlation of total protein intensity for single-step Azo, dual-step Decell, and dual-step Azo extraction replicates (Fig. 2*C*). Pearson correlation coefficients for single-step Azo extracts fell between 0.962 and 0.976 (supplemental Fig. S2*A*). For the dual-step protocol, Pearson correlation coefficients were between 0.954 and 0.968 for Decell extracts (supplemental Fig. S2*B*) and 0.953 and 0.970 for Azo extracts (supplemental Fig. S2*C*).

Our single-step Azo extraction demonstrates reproducibility in the total proteins and matrisome protein identifications across extracts. Furthermore, the reproducibility of the single-step protocol is consistent with the extraction reproducibility observed with our previous dual-step method (21).

### Comparable Matrisome Protein Identifications in Single-Step *versus* Dual-Step Azo Extracts

**Fig. 3:**
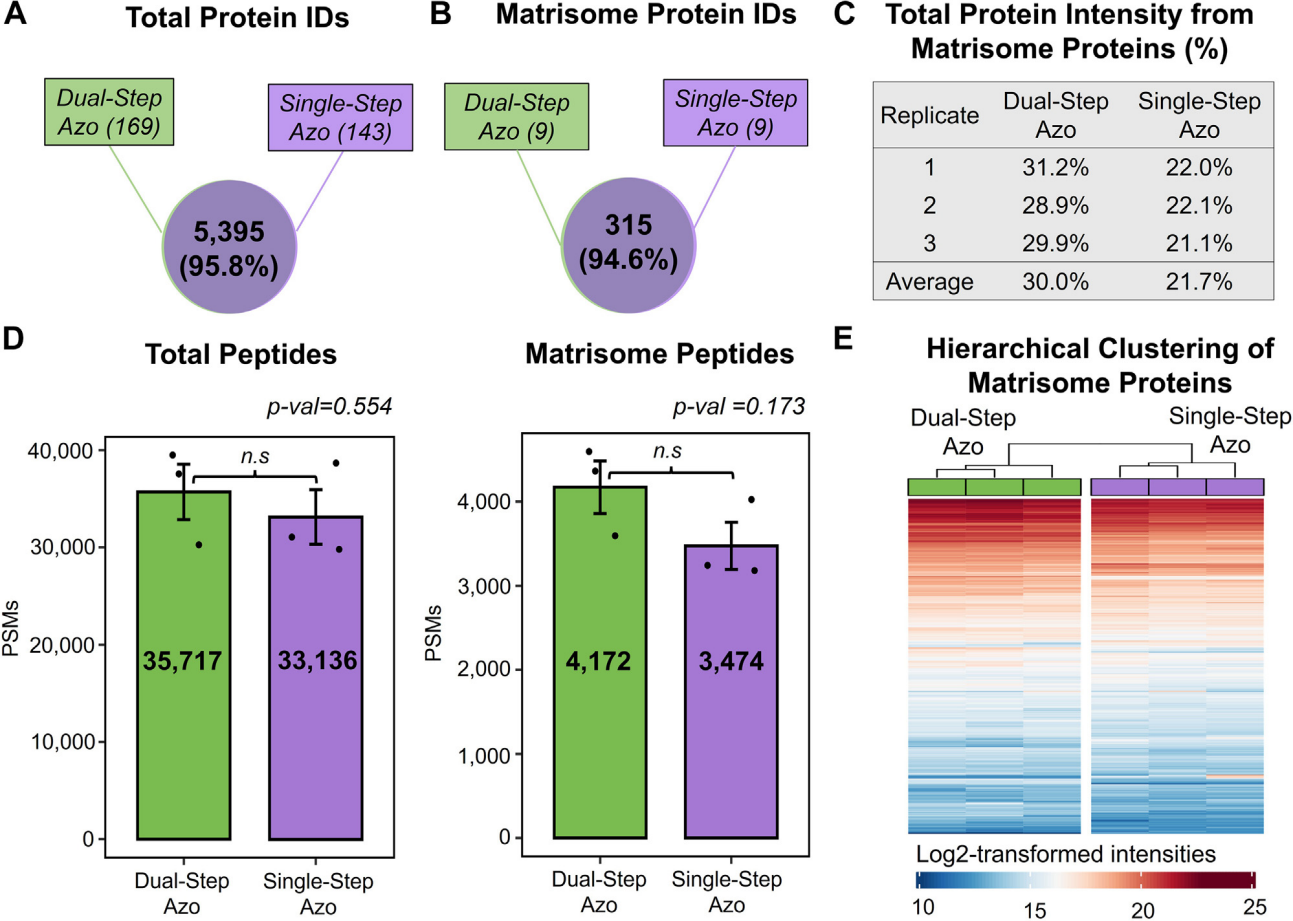
**Protein identifications from single-step Azo extracts are comparable with dual-step Azo extracts.***A*, Venn diagram shows the total number of unique protein identifications in common between dual-step Azo and single-step Azo extracts. *B*, Venn diagram demonstrates overlap in matrisome proteins quantified in dual-step Azo and single-step Azo extracts. *C*, The percentage of total protein intensity resulting from matrisome proteins calculated for each extraction replicate. *D*, The number of total peptides and the number of matrisome peptides identified in each extraction. *E*, Hierarchical clustering analysis of the matrisome proteins identified across n = 3 dual-step Azo and n = 3 single-step Azo extraction replicates. Matrisome proteins are arranged from highest to lowest average Log2-transformed intensity across all six replicates.

The total number of proteins identified in single-step Azo extracts was compared with the proteins identified in dual-step Azo extracts. Of the 5488 proteins identified in dual-step Azo extracts and the 5538 identified in single-step Azo extracts, 5395 proteins were detected in both groups, corresponding to a 95.8% overlap in protein identifications (Fig. 3*A*). 324 total matrisome proteins were identified in both dual-step Azo and single-step Azo extracts. 315 matrisome proteins were quantified in both dual-step and single-step Azo extracts, resulting in a 94.6% overlap in matrisome protein identifications (Fig. 3*B*). The total protein intensity resulting from matrisome proteins was calculated for each extraction replicate by summing protein intensities associated with matrisome proteins and dividing by the sum of total protein intensities. The average intensity resulting from matrisome proteins in dual-step Azo extracts was 30.0% while the average for single-step Azo extracts was 21.7% (Fig. 3*C*). A decellularization extraction prior to Azo solubilization depletes abundant intracellular and cytosolic proteins, so it is expected that matrisome proteins will make up a greater percentage of protein intensity in dual-step Azo extracts compared with single-step Azo extracts.

An average of 35,717 total peptides were detected in dual-step Azo extracts and 33,136 in single-step Azo extracts. An average of 4172 matrisome peptides was detected in dual-step Azo extracts and 3474 in single-step Azo extracts (Fig. 3*D*). The average numbers of total peptides and matrisome-specific peptides identified in single-step Azo fractions were lower than dual-step Azo extracts, and a student’s *t*-test was performed to assess whether this difference was significant. The unadjusted *p*-values for dual-step and single-step Azo extracts were calculated as 0.554 for total peptides and 0.173 for matrisome peptides, indicating the difference in the number of peptides detected in the two different extraction types fails to reach statistical significance. Hierarchical clustering analysis of the matrisome proteins detected in dual-step Azo and single-step Azo extracts shows proteins ordered based on average intensities across all six extracts, indicating relative ranked abundance of matrisome proteins is conserved in the single-step Azo extracts relative to the dual-step Azo extracts (Fig. 3*E*). Quantitative reproducibility was assessed for dual-step and single-step Azo extracts using the coefficient of variation. Following filtering and imputation, protein intensities were Log_2_-transformed to achieve a normal distribution of values. For both dual-step and single-step Azo total protein intensities (supplemental Fig. S3*A*) and matrisome protein intensities (supplemental Fig. S3*B*), the median coefficient of variation is less than 2%, demonstrating quantitative reproducibility of total and matrisome protein quantitation using both protein extraction methods. Gene ontology-cellular component (GOCC) overrepresentation analysis of the total proteins detected in single-step Azo extracts include both extracellular and intracellular components (supplemental Fig. S4*A*). The six GOCC terms with the highest fold enrichment are related to the extracellular region and include extracellular exosomes, extracellular vesicles, extracellular organelles, extracellular membrane-bounded organelles, vesicles, and the extracellular space. GOCC overrepresentation analysis of the total proteins identified in dual-step Azo extracts shows distinct similarities in the GO terms and gene counts to single-step Azo GOCC analysis (supplemental Fig. S4*B*). This demonstrates the matrisome coverage achieved with the single-step Azo extraction protocol is comparable with the coverage observed with the dual-step Azo extracts.

The comparison between dual-step Azo and single-step Azo extracts demonstrates matrisome protein identifications achieved with a single-step Azo extraction significantly overlap with those detected in the dual-step Azo extracts. Dual-step Azo extracts reflect larger matrisome peptide counts and greater protein intensity attributed to matrisome proteins compared with single-step Azo extracts due to the decellularization step which depletes abundant intracellular proteins; however, matrisome protein identifications in single-step Azo extracts remain comparable to dual-step Azo extracts despite the absence of a decellularization step.

### Total Matrisome Protein Identifications From Dual-Step Extracts are Similar to Single-Step Azo Extracts

**Fig. 4:**
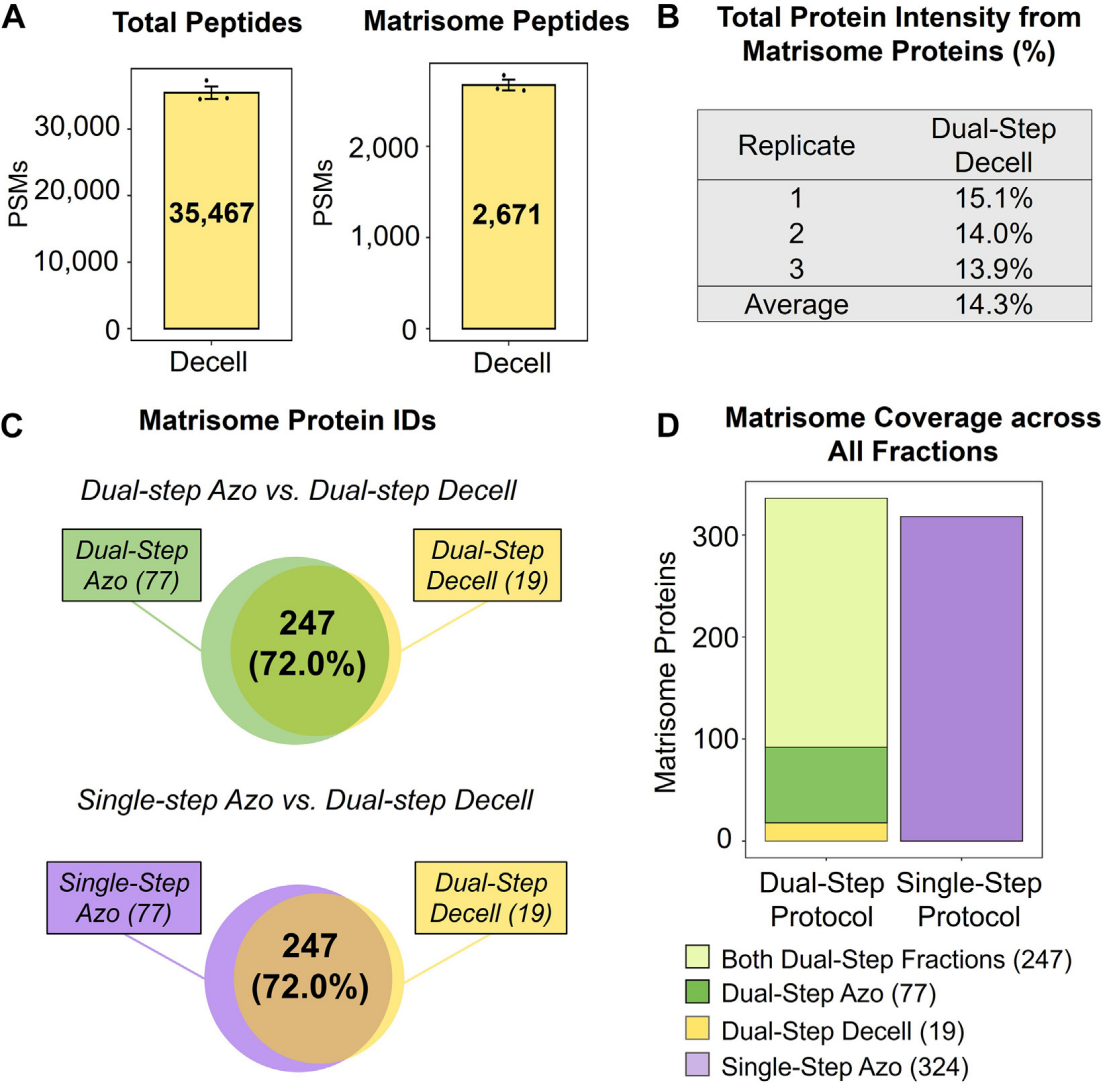
**Total matrisome protein identifications from dual-step Decell and dual-step Azo extracts are comparable to single-step Azo extracts.***A*, Total unique peptides and matrisome peptides identified in the dual-step Decell extracts across three extraction replicates. *B*, The percentage of total protein intensity resulting from matrisome proteins calculated for each extraction replicate. *C*, Venn diagrams demonstrate the overlap in matrisome protein identifications between dual-step Azo and dual-step Decell, as well as the overlap between single-step Azo and dual-step Decell extracts. *D*, Summary of matrisome protein coverage across all extracts.

Protein identifications in the dual-step Decell extracts were compared to single-step and dual-step Azo extracts. An average of 35,467 total peptides were detected across n = 3 dual-step Decell extraction replicates. There was an average of 2671 matrisome peptides detected in dual-step Decell extracts (Fig. 4*A*). The total protein intensity from matrisome proteins in Decell extracts was calculated for each extraction replicate, and an average of 14.3% of protein intensity was a result of matrisome protein identifications (Fig. 4*B*). Compared to dual-step Azo (30.0%) and single-step Azo (21.7%) (Fig. 3*B*), matrisome protein identifications represent less of the total protein intensity in Decell extracts. A lower percentage of total protein intensity resulting from matrisome proteins is expected for the Decell extract because many ECM proteins are not solubilized in the decellularization step. Overlap in total protein and matrisome protein identifications were assessed between dual-step Azo and dual-step Decell as well as single-step Azo and dual-step Decell extracts. Comparing dual-step Azo and Decell total protein identifications, 4488 proteins were identified in both Azo and Decell extracts, resulting in a 68.4% overlap in identifications (supplemental Fig. S5*A*). There were 4560 proteins identified in common between single-step Azo and dual-step Decell extracts with a 69.7% overlap (supplemental Fig. S5*B*). Comparing dual-step Azo and Decell fractions, 247 matrisome proteins were identified in both Azo and Decell extracts, corresponding to a 72.0% overlap. There were 77 matrisome proteins unique to the dual-step Azo extracts, including fibrillar collagen species which contribute to the architecture of the lung as well as network-forming collagens which form collagen networks in the basement membrane for facilitating cellular processes (33, 34, 35). Several glycoproteins were also uniquely identified in the dual-step Azo extracts compared with the dual-step Decell, including thrombospondin-4 which has recently been implicated in pulmonary arterial hypertension (36). Additionally, several disintegrins and metalloproteinases which are known to be dysregulated in lung cancer and fibrotic diseases were detected in dual-step Azo extracts but absent from Decell extracts (37, 38). Comparing single-step Azo and dual-step Decell extracts, there were 247 matrisome proteins identified in common between the two conditions, resulting in a 72.0% overlap (Fig. 4*C*). There were also 77 matrisome proteins unique to the single-step Azo extracts compared with the dual-step Decell extracts including fibrillar and non-fibrillar collagens as well as semaphorins which have been linked to multiple cancer processes and more recently, to idiopathic pulmonary fibrosis (39, 40).

Based on comparisons between the Decell and dual-step and single-step Azo extracts, both Azo extracts demonstrate more comprehensive coverage of the matrisome. The overlap in matrisome proteins identified in the dual-step Decell and Azo extracts compared with the overlap in identifications across single-step Azo and Decell extracts further demonstrates the similarities in matrisome coverage between dual-step and single-step Azo extracts. In summary, 343 matrisome proteins were identified with the dual-step extraction protocol across Decell and Azo extracts. 247 matrisome proteins were quantified in both extracts while 77 were unique to dual-step Azo extracts and 19 unique to the Decell extracts. A total of 324 unique matrisome proteins were quantified in single-step Azo extracts (Fig. 4*D*). Between single-step and dual-step protocols, 19 additional matrisome proteins were identified with the dual-step extraction method compared to the single-step protocol.

### Single-Step Azo Extraction Matrisome Coverage

**Fig. 5:**
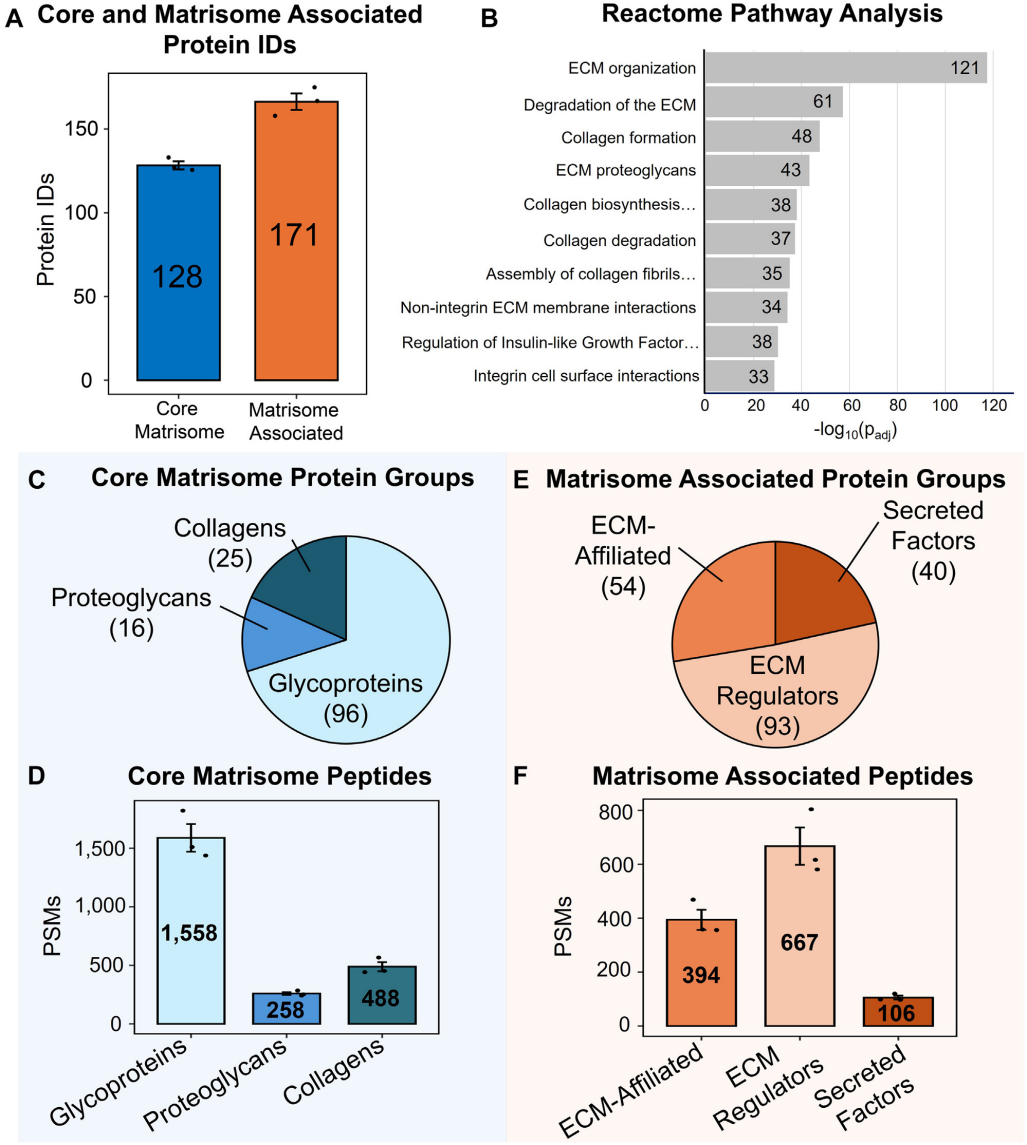
**Single-step Azo extract matrisome coverage.***A*, The average number of core and matrisome-associated proteins identified in the single-step Azo extracts across three extraction replicates. *B*, STRING protein-protein interaction analysis demonstrates the most significantly enriched Reactome Pathways from the matrisome proteins identified in single-step Azo extracts. The number of protein identifications associated with the pathway is shown in each bar, and the pathways are sorted by -log10(FDR-adjusted *p*-value). *C*, Matrisome protein identifications consist of the core matrisome divided into glycoproteins, proteoglycans, and collagens. *D*, Number of peptides corresponding to glycoproteins, proteoglycans, and collagens. *E*, Matrisome-associated proteins, consisting of ECM-affiliated proteins, ECM regulators, and secreted factors. *F*, Number of peptides mapping to ECM-affiliated proteins, ECM regulators, and secreted factors.

Our single-step protein extraction method efficiently solubilizes the ECM with a single Azo extraction. An average of 128 core matrisome proteins and 171 matrisome-associated proteins were identified across n = 3 single-step Azo extraction replicates (Fig. 5*A*). A Reactome Pathway analysis was performed for the 324 matrisome proteins detected in single-step Azo extraction replicates. The top three most significantly enriched pathways included extracellular matrix organization (121 gene identifications), degradation of the extracellular matrix (61 gene identifications), and collagen formation (48 gene identifications) (Fig. 5*B*). Comparing our Reactome Pathway analysis to the analysis performed in our previous dual-step method, we observe the enriched pathways to be nearly identical (21). Core matrisome protein identifications were further broken down into glycoproteins, proteoglycans, and collagens in the same manner as previously reported (21). In single-step Azo extracts, 96 glycoproteins, 16 proteoglycans, and 25 collagen species were quantified (Fig. 5*C*). An average of 1558 unique glycoprotein peptides, 258 proteoglycan peptides, and 488 collagen peptides were detected in single-step Azo extracts (Fig. 5*D*). The core matrisome proteins identified in single-step Azo extracts are summarized in supplemental Table S1. Matrisome-associated protein identifications were broken down into ECM-affiliated proteins, ECM regulators, and secreted factors. Among the matrisome-associated proteins identified, 54 were ECM-affiliated proteins, 93 were ECM regulators, and 40 were secreted factors (Fig. 5*E*). An average of 394 ECM-affiliated peptides, 667 ECM regulator peptides, and 106 secreted factor peptides were detected across single-step Azo extraction replicates (Fig. 5*F*). The matrisome-associated proteins identified in single-step Azo extracts are summarized in supplemental Table S2. In dual-step Decell fractions, an average of 112 core matrisome and 143 matrisome-associated proteins were identified in dual-step extraction replicates (supplemental Fig. S6*A*). Core matrisome identifications included 81 glycoproteins, 14 proteoglycans, and 20 collagens, and matrisome-associated identifications included 45 ECM-affiliated proteins, 76 ECM regulators, and 26 secreted factors (supplemental Fig. S6, *B* and *C*). Dual-step Azo matrisome protein identifications included an average of 130 core matrisome and 175 matrisome-associated proteins (supplemental Fig. S7*A*). Core matrisome identifications were broken down into 95 glycoproteins, 14 proteoglycans, and 24 collagen species (supplemental Fig. S7*B*). Matrisome-associated proteins included 50 ECM-affiliated proteins, 94 ECM regulators, and 41 secreted factors (supplemental Fig. S7*C*). Comparing Decell matrisome identifications to dual-step and single-step Azo extracts, a greater number of unique glycoproteins, collagens, ECM regulators, and secreted factors are observed in Azo extracts. The matrisome protein identifications in dual-step and single-step Azo extracts significantly overlap, demonstrating our single-step Azo extraction protocol provides matrisome coverage comparable to our dual-step method.

**Fig. 6:**
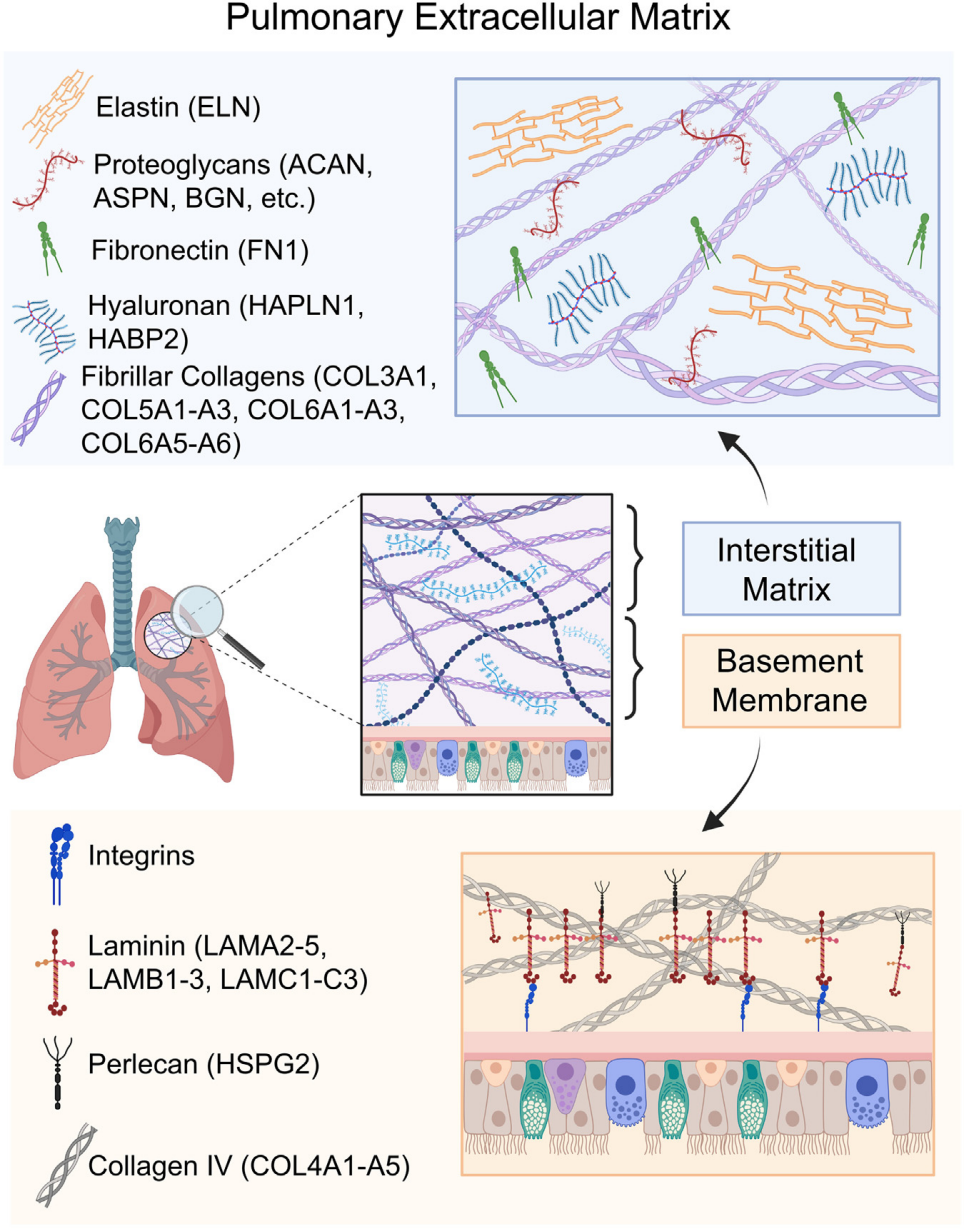
**Representative matrisome proteins identified using single-step Azo protocol including interstitial matrix and basement membrane proteins.** The gene family for the ECM proteins detected in single-step Azo extracts is included next to each protein type. A comprehensive list of the core and matrisome associated proteins detected in single-step Azo extracts is included in the supplementary information (supplemental Tables S1 and S2). Aspects of this figure were generated using BioRender.

In summary, we detected core and matrisome-associated proteins which are major components of the pulmonary ECM, including basement membrane and interstitial matrix proteins. Figure 6 provides an overview of several matrisome proteins detected with our single-step Azo extraction which are critical to lung biology and the pathogenesis of lung diseases (10, 11). We identified important structural proteins of the interstitial matrix, such as elastin which provides elastic recoil, and fibrillar collagens which provide structural scaffolding and mechanical stability in the lung (35, 41, 42). Additionally, we detected the cell adhesion protein fibronectin and hyaluronan-binding proteins which have been implicated in pulmonary diseases (43, 44). We also identified basement membrane proteins including type IV collagen which provides a network for binding glycoproteins, proteoglycans, and various growth factors (35, 45). Laminins, a type of cell adhesion protein, and perlecan, a heparan sulfate proteoglycan, are additional key components of the basement membrane involved in lung development which were detected in our single-step Azo extracts (46, 47). Comprehensive lists of the core matrisome and matrisome-associated proteins identified with the single-step Azo extraction method are included in supplemental Tables S1 and S2.

When comparing the matrisome protein coverage achieved with this single-step Azo protocol to the previous dual-step results, we observed significant overlap in core and matrisome-associated protein identifications (21). The dual-step method previously reported the detection of fibrillar collagens, including types I, II, III, V, and XI in the Azo extracts as well as other core matrisome proteins such as elastin. We reproducibly recovered the same collagen species in this study and observed similar core matrisome identifications as those reported in the previous paper using the dual-step method (21). The classification of core matrisome identifications into collagens, proteoglycans, and glycoproteins and matrisome-associated identifications into ECM regulators, ECM-affiliated proteins, and secreted factors in this study here is comparable with the matrisome proteins reported in the previous dual-step method (21). Overall, we observe consistency in the identification of matrisome proteins across experiments, underscoring the robustness and reproducibility of the Azo-enabled extraction protocols.

## Conclusion

We have developed a reproducible, high-throughput single-step Azo-enabled protocol for the quantitative analysis of matrisome proteins from lung tissue which can be broadly applied to ECM proteomics applications in other tissue types. This method effectively solubilizes both core and matrisome-associated proteins, achieving coverage comparable to our dual-step extraction. By streamlining the process to a single step, we significantly enhance throughput, reducing the time and labor required for protein extraction, sample preparation, and data analysis. This approach simplifies relative protein quantification from a single extract per biological sample, facilitating large-scale discovery experiments in ECM biology and disease across various tissue types, particularly in studies requiring large biological cohorts.

## Data Availability

The mass spectrometry proteomics data have been deposited to the ProteomeXchange Consortium *via* the PRIDE partner repository with the data set identifier PXD057809 and the MassIVE repository with identifier MSV000096393.

## Supplemental data

This article contains supplemental data.

## Conflict of interest

The authors declare the following financial interests/personal relationships which may be considered as potential competing interests: Y. G. is a co-inventor on a patent that covers the detergent Azo (US Patent No: US11,567,085 B2). Funding for this work was provided by AbbVie, Inc. F. W., S. L., K. K, Y. H., and Y. T. are employees of AbbVie, Inc. AbbVie participated in the design and conduct of this research. AbbVie participated in the interpretation of data, review, and approval of the publication. AbbVie takes no position on the tools and technologies shown. All statements regarding tools and technologies are the opinions of the authors.

## Supplementary Data

Supplementary file 5

ECM_methods_revised_SI_figures_R2
