# Supplementary material for: A Single-Step Protein Extraction for Lung Extracellular Matrix Proteomics Enabled by the Photocleavable Surfactant Azo and timsTOF Pro
Source: Mol Cell Proteomics. 2025 Mar 17;24(8):100950. doi: 10.1016/j.mcpro.2025.100950 (PMC12535824; doi:10.1016/j.mcpro.2025.100950)
Supplement: ECM_methods_revised_SI_figures_R2 [file mmc6.pdf]

Supplementary Information

A Single-Step Protein Extraction for Lung Extracellular Matrix Proteomics Enabled by the Photocleavable Surfactant Azo and timsTOF Pro

Anna G. Towler[1], Andrew J. Perciaccante[1], Timothy J. Aballo[2], Yanlong Zhu[2,3], Fei Wang[4], Sarah Lloyd[5], Kuniko Kadoya[6], Yupeng He*[5], Yu Tian*[4], Ying Ge*[1,2,3]

[1] Department of Chemistry, University of Wisconsin-Madison, Madison, WI 53706, USA

[2] Molecular and Cellular Pharmacology Training Program, University of Wisconsin-Madison, Madison, WI 53705, USA

[3] Human Proteomics Program, School of Medicine and Public Health, University of Wisconsin-Madison, Madison, WI 53705, USA

[4] Quantitative Translational & ADME Science, AbbVie Bioresearch Center, Worcester, MA 01605.

[5] Discovery Immunology, Pharmacology and Pathology, AbbVie, Inc., North Chicago, IL 60064.

[6] Allergan Aesthetics, an AbbVie company, 2525 Dupont Drive, Irvine, CA 92612 USA

*To whom correspondence should be addressed: Dr. Ying Ge, 1111 Highland Ave., WIMR II 8551, Madison, WI 53705. Email: ying.ge@wisc.edu. Tel: 608-265-4744. Dr. Yu Tian, 100 Research Dr. Worcester, MA, 01605. Email: yu.tian@abbvie.com. Tel: 508-688-3561. Dr. Yupeng He, 1 North Waukegan Road, North Chicago, IL 60064. Email: yupeng.he@abbvie.com. Tel: 847-936-6203.

Table of Contents

Supplemental Figures

- Figure S1. Reproducibility of single-step and dual-step extraction methods.
- Figure S2. Pearson correlation plots for single-step and dual-step extraction methods.
- Figure S3. Coefficient of variation for Log2-transformed protein intensities
- Figure S4. Gene ontology- cellular component (GOCC) overrepresentation analysis of total proteins identified in single-step Azo and dual-step Azo extracts.
- Figure S5. Venn diagrams comparing overlap in total protein identifications between dual-step Azo and dual-step Decell as well as single-step Azo and dual-step Decell extracts.
- Figure S6. Dual-step Decell matrisome coverage.
- Figure S7. Dual-step Azo matrisome coverage.

Supplemental Tables

- Table S1. Core matrisome proteins identified in single-step Azo extracts.
- Table S2. Matrisome associated proteins identified in single-step Azo extracts.

Supplementary Files

- Supplementary File 1: diaPASEF Parameters
- Supplementary File 2: DIA-NN Protein Group Output File for Azo Extracts with Unique Peptides
- Supplementary File 3: DIA-NN Protein Group Output File for Decell Extracts with Unique Peptides

- Supplementary File 4: DIA-NN Peptide Output File for Azo Extracts
- Supplementary File 5: DIA-NN Peptide Output File for Decell Extracts

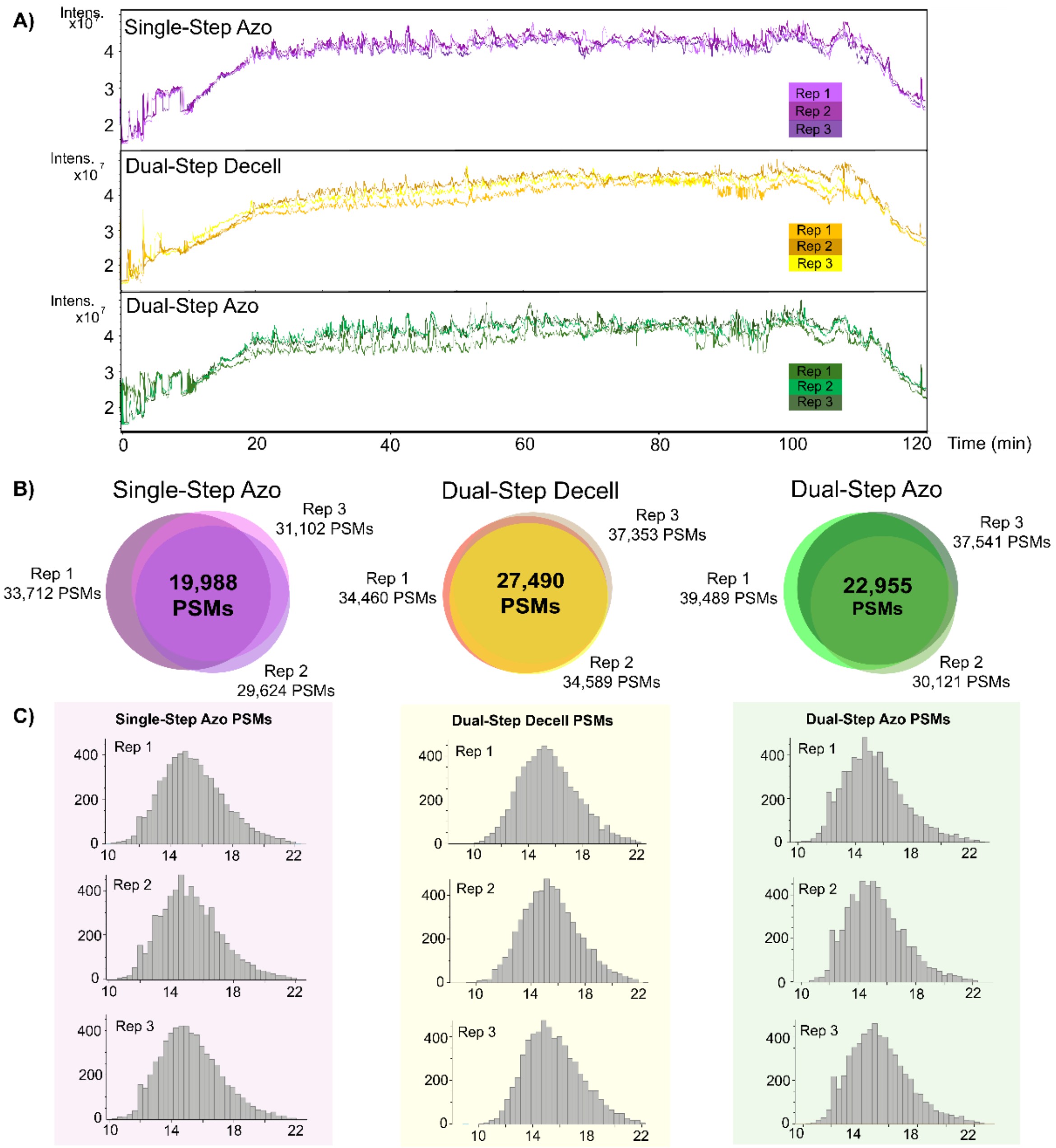

Figure S1: Reproducibility of single-step and dual-step extraction methods. A) Total ion chromatograms (MS1) are overlaid for n=3 single-step Azo (purple), dual-step Decell (yellow), and dual-step Azo (green) extraction replicates. 200 ng peptides were injected for each replicate. **B)** Venn diagrams demonstrate overlap in the number of peptide spectral matches (PSMs) identified in n=3 extraction replicates for each condition. **C)** Histograms of Log2-transformed peptide intensities.

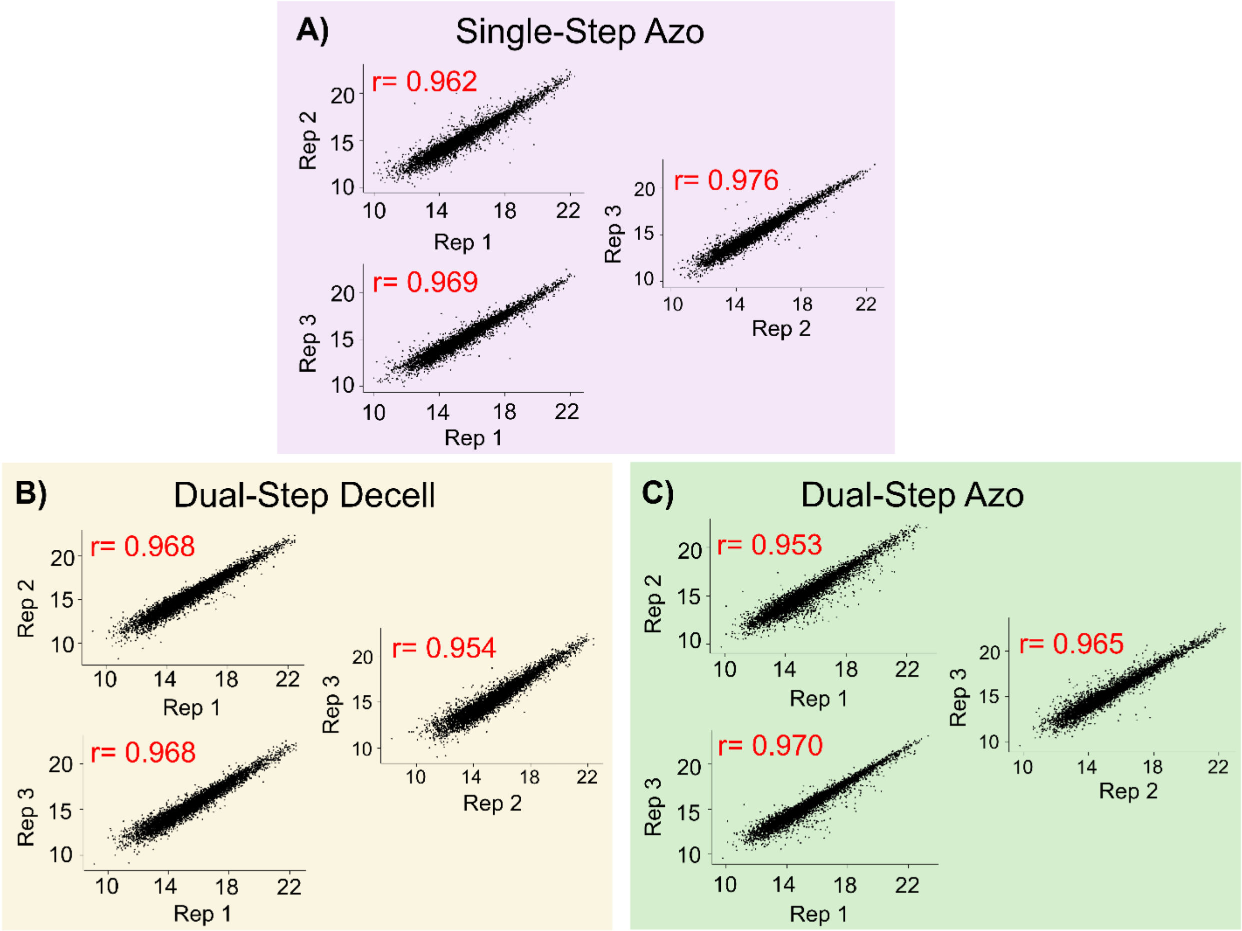

Figure S2: Pearson correlation plots demonstrate high correlation between replicates. Pearson correlation coefficients for **A**) single-step Azo, **B**) dual-step Decell, and **C**) dual-step Azo extraction replicates.

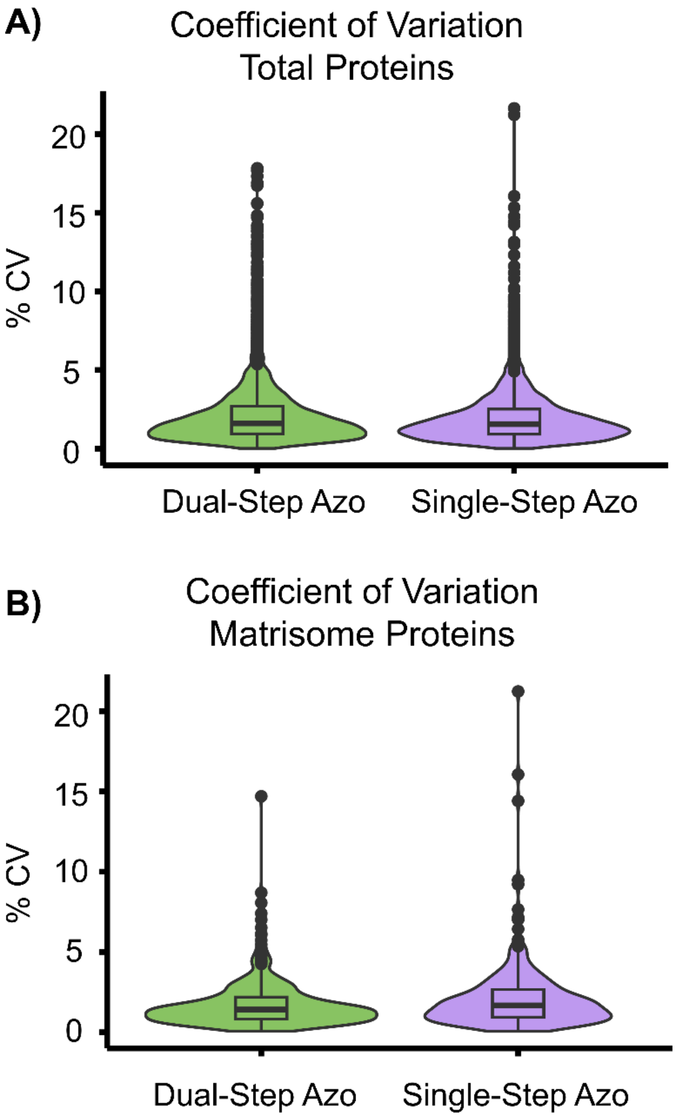

Figure S3: Coefficient of variation for dual-step and single-step Azo extracts calculated from Log2-transformed protein intensities after filtering and imputation. **A)** Coefficient of variation calculated from total protein intensities in dual-step and single-step Azo extracts. **B)** Coefficient of variation calculated from matrisome protein intensities in dual-step and single-step Azo extracts.

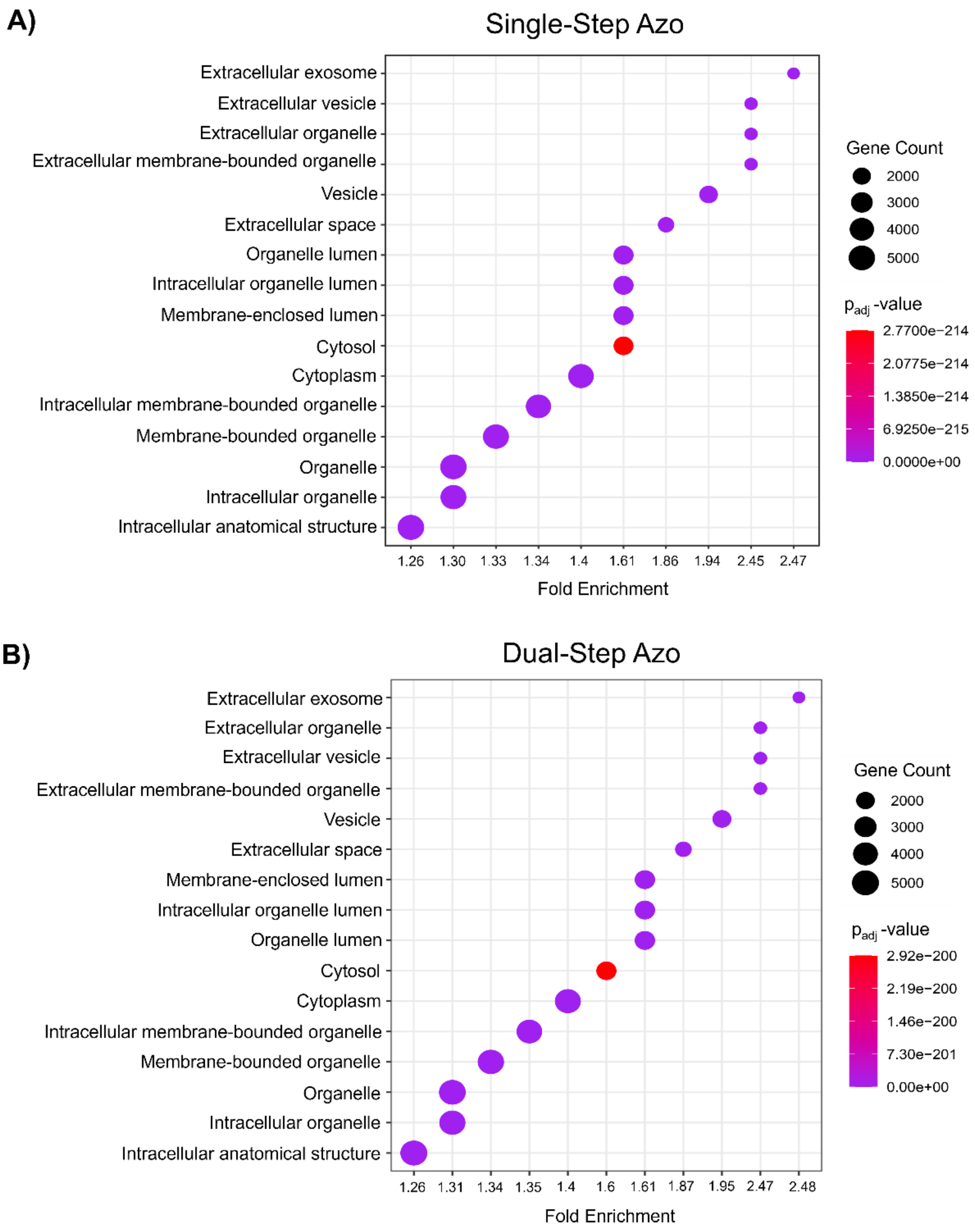

Figure S4: Gene ontology- cellular component (GOCC) overrepresentation analysis of the total proteins identified in single-step Azo and dual-step Azo extracts. Top 16 GOCC terms with the highest fold enrichment from proteins identified in **(A)** single-step Azo extracts and **(B)** dual-step Azo extracts. Circle size indicates the number of genes mapped to a GO term from proteomics data. Circle color indicates adjusted p-value.

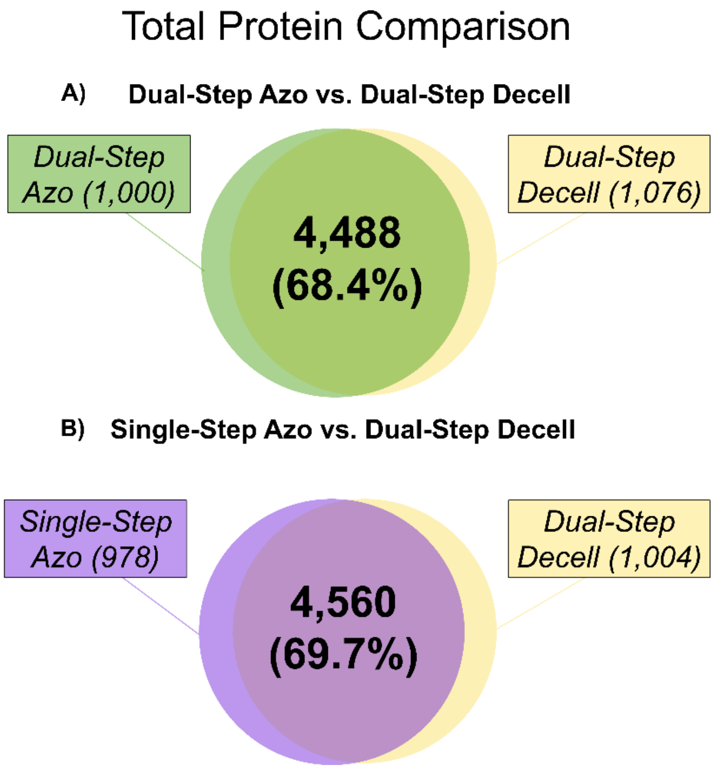

Figure S5: Total protein identifications between dual-step Decell and Azo extracts. **A)** Venn diagram demonstrates the overlap in total proteins identified in dual-step Azo and dual-step Decell extracts. **B)** Venn diagram shows total proteins identified in common between single-step Azo and dual-step Decell extracts.

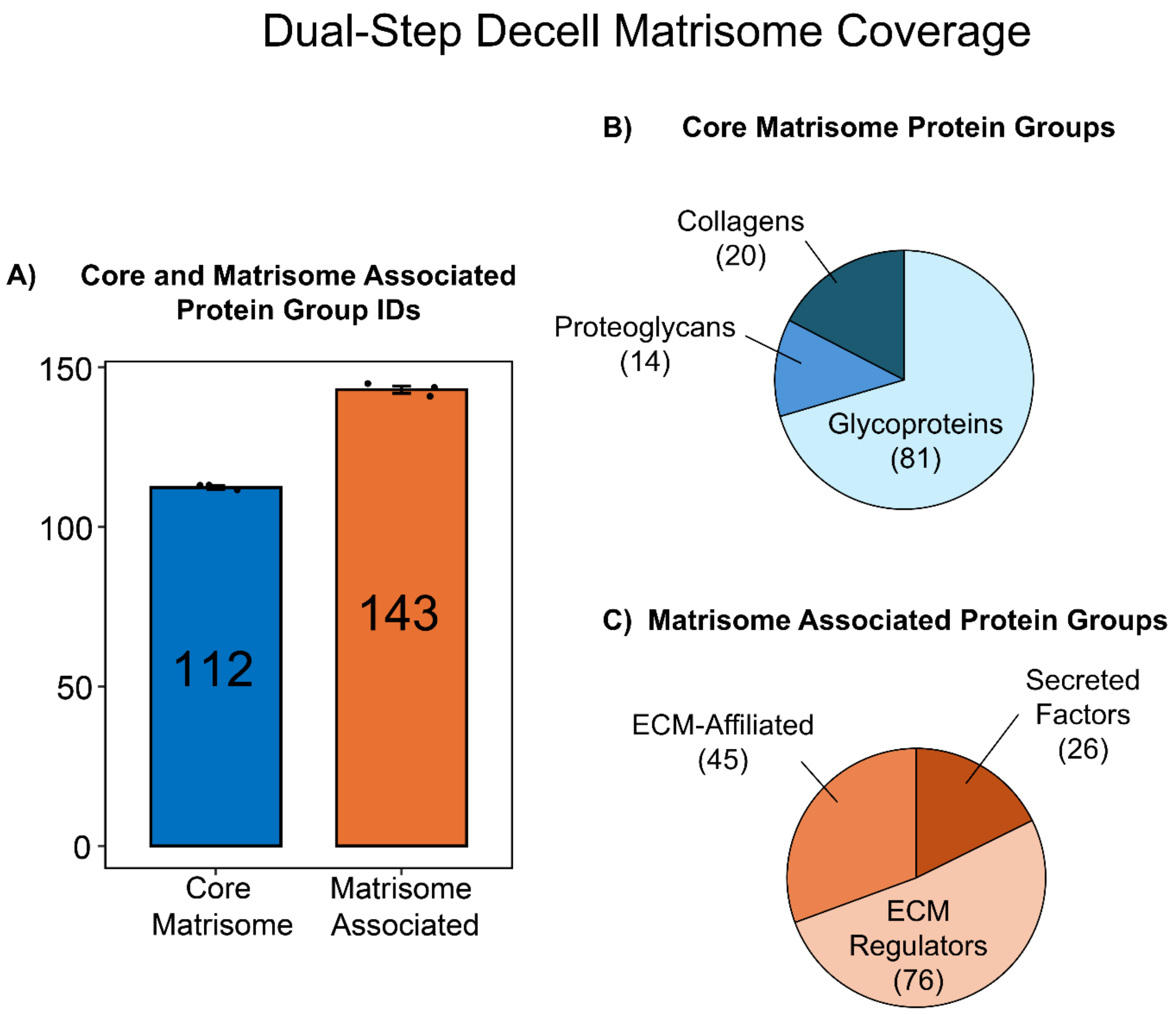

Figure S6: Dual-step Decell matrisome coverage. A) Number of core matrisome and matrisome-associated proteins identified in dual-step Decell extracts in n=3 extraction replicates. **B)** Core matrisome protein identifications divided into glycoproteins, proteoglycans, and collagens. **C)** Matrisome-associated proteins, consisting of ECM-affiliated proteins, ECM regulators, and secreted factors.

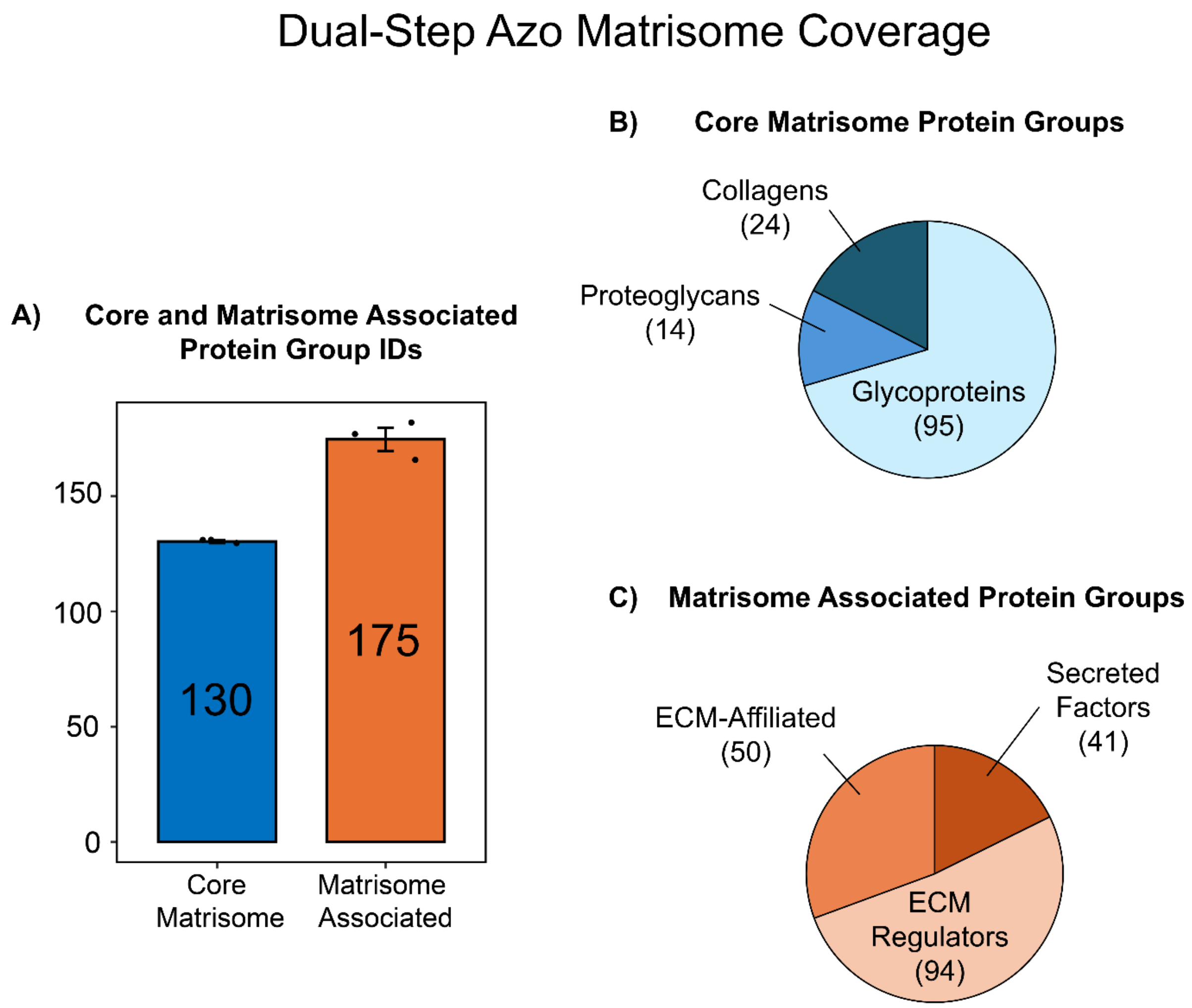

Figure S7: Dual-step Azo matrisome coverage. A) Number of core matrisome and matrisome-associated proteins identified in dual-step Azo extracts in n=3 extraction replicates. **B)** Core matrisome protein identifications divided into glycoproteins, proteoglycans, and collagens. **C)** Matrisome-associated proteins, consisting of ECM-affiliated proteins, ECM regulators, and secreted factors.

Table S1: Core matrisome proteins identified in single-step Azo extracts. 137 of the 324 unique matrisome proteins identified across 3 single-step Azo extraction replicates were core matrisome proteins.

UniProt ID	Genes	Protein Name
Q7Z7G0	ABI3BP	Target of Nesh-SH3
P16112	ACAN	Aggrecan core protein
Q15848	ADIPOQ	Adiponectin
Q8IUX7	AEBP1	Adipocyte enhancer-binding protein 1
O00468	AGRN	Agrin
Q9BXN1	ASPN	Asporin
P21810	BGN	Biglycan
Q8N8U9	BMPER	BMP-binding endothelial regulator protein
O00622	CCN1	CCN family member 1
O76076	CCN5	CCN family member 5
O75339	CILP	Cartilage intermediate layer protein 1
Q99715	COL12A1	Collagen alpha-1(XII) chain
Q05707	COL14A1	Collagen alpha-1(XIV) chain
P39059	COL15A1	Collagen alpha-1(XV) chain
Q07092	COL16A1	Collagen alpha-1(XVI) chain
P39060	COL18A1	Collagen alpha-1(XVIII) chain
P02452	COL1A1	Collagen alpha-1(I) chain
P08123	COL1A2	Collagen alpha-2(I) chain
Q96P44	COL21A1	Collagen alpha-1(XXI) chain
P02458	COL2A1	Collagen alpha-1(II) chain
P02461	COL3A1	Collagen alpha-1(III) chain
P02462	COL4A1	Collagen alpha-1(IV) chain
P08572	COL4A2	Collagen alpha-2(IV) chain
Q01955	COL4A3	Collagen alpha-3(IV) chain
P53420	COL4A4	Collagen alpha-4(IV) chain
P29400	COL4A5	Collagen alpha-5(IV) chain
P20908	COL5A1	Collagen alpha-1(V) chain
P05997	COL5A2	Collagen alpha-2(V) chain
P25940	COL5A3	Collagen alpha-3(V) chain
P12109	COL6A1	Collagen alpha-1(VI) chain
P12110	COL6A2	Collagen alpha-2(VI) chain
P12111	COL6A3	Collagen alpha-3(VI) chain
A8TX70	COL6A5	Collagen alpha-5(VI) chain
A6NMZ7	COL6A6	Collagen alpha-6(VI) chain
Q02388	COL7A1	Collagen alpha-1(VII) chain
P27658	COL8A1	Collagen alpha-1(VIII) chain

Q96HD1	CRELD1	Protein disulfide isomerase CRELD1
Q6UXH1	CRELD2	Protein disulfide isomerase CRELD2
Q9H0B8	CRISPLD2	Cysteine-rich secretory protein LCCL domain-containing 2
Q96CG8	CTHRC1	Collagen triple helix repeat-containing protein 1
P07585	DCN	Decorin
Q9UGM3	DMBT1	Scavenger receptor cysteine-rich domain-containing protein DMBT1
Q07507	DPT	Dermatopontin
Q16610	ECM1	Extracellular matrix protein 1
O43854	EDIL3	EGF-like repeat and discoidin I-like domain-containing protein 3
Q12805	EFEMP1	EGF-containing fibulin-like extracellular matrix protein 1
O95967	EFEMP2	EGF-containing fibulin-like extracellular matrix protein 2
P15502	ELN	Elastin
Q9Y6C2	EMILIN1	EMILIN-1
Q9BXX0	EMILIN2	EMILIN-2
Q99645	EPYC	Epiphycan
P23142	FBLN1	Fibulin-1
P98095	FBLN2	Fibulin-2
Q9UBX5	FBLN5	Fibulin-5
P35555	FBN1	Fibrillin-1
P35556	FBN2	Fibrillin-2
P02671	FGA	Fibrinogen alpha chain
P02675	FGB	Fibrinogen beta chain
P02679	FGG	Fibrinogen gamma chain
Q14314	FGL2	Fibroleukin
Q06828	FMOD	Fibromodulin
P02751	FN1	Fibronectin
Q14393	GAS6	Growth arrest-specific protein 6
P10915	HAPLN1	Hyaluronan and proteoglycan link protein 1
Q96RW7	HMCN1	Hemicentin-1
P98160	HSPG2	Basement membrane-specific heparan sulfate proteoglycan core protein
P35858	IGFALS	Insulin-like growth factor-binding protein complex acid labile subunit
P18065	IGFBP2	Insulin-like growth factor-binding protein 2
P22692	IGFBP4	Insulin-like growth factor-binding protein 4
Q16270	IGFBP7	Insulin-like growth factor-binding protein 7
P24043	LAMA2	Laminin subunit alpha-2
Q16787	LAMA3	Laminin subunit alpha-3
Q16363	LAMA4	Laminin subunit alpha-4
O15230	LAMA5	Laminin subunit alpha-5

P07942	LAMB1	Laminin subunit beta-1
P55268	LAMB2	Laminin subunit beta-2
Q13751	LAMB3	Laminin subunit beta-3
P11047	LAMC1	Laminin subunit gamma-1
Q13753	LAMC2	Laminin subunit gamma-2
Q9Y6N6	LAMC3	Laminin subunit gamma-3
P02750	LRG1	Leucine-rich alpha-2-glycoprotein
Q14766	LTBP1	Latent-transforming growth factor beta-binding protein 1
Q14767	LTBP2	Latent-transforming growth factor beta-binding protein 2
Q8N2S1	LTBP4	Latent-transforming growth factor beta-binding protein 4
P51884	LUM	Lumican
P21941	MATN1	Cartilage matrix protein
O00339	MATN2	Matrilin-2
P55081	MFAP1	Microfibrillar-associated protein 1
P55001	MFAP2	Microfibrillar-associated protein 2
P55083	MFAP4	Microfibril-associated glycoprotein 4
Q13361	MFAP5	Microfibrillar-associated protein 5
Q08431	MFGE8	Lactadherin
P08493	MGP	Matrix Gla protein
Q13201	MMRN1	Multimerin-1
Q9H8L6	MMRN2	Multimerin-2
Q9NR99	MXRA5	Matrix-remodeling-associated protein 5
Q8TB73	NDNF	Protein NDNF
P14543	NID1	Nidogen-1
Q14112	NID2	Nidogen-2
Q6UXI9	NPNT	Nephronectin
Q9HB63	NTN4	Netrin-4
P20774	OGN	Mimecan
O95428	PAPLN	Papilin
Q15113	PCOLCE	Procollagen C-endopeptidase enhancer 1
Q9UKZ9	PCOLCE2	Procollagen C-endopeptidase enhancer 2
Q7Z5L7	PODN	Podocan
Q15063	POSTN	Periostin
P51888	PRELP	Prolargin
P13727	PRG2	Bone marrow proteoglycan
Q9Y2Y8	PRG3	Proteoglycan 3
Q92954	PRG4	Proteoglycan 4
Q92626	PXDN	Peroxidasin homolog
Q8IVN8	SBSPON	Somatomedin-B and thrombospondin type-1 domain-containing protein
O94813	SLIT2	Slit homolog 2 protein
O75094	SLIT3	Slit homolog 3 protein

P09486	SPARC	SPARC
Q14515	SPARCL1	SPARC-like protein 1
Q9HCB6	SPON1	Spondin-1
Q9BUD6	SPON2	Spondin-2
P78539	SRPX	Sushi repeat-containing protein SRPX
O60687	SRPX2	Sushi repeat-containing protein SRPX2
Q4LDE5	SVEP1	Sushi, von Willebrand factor type A, EGF and pentraxin domain-containing protein 1
Q15582	TGFBI	Transforming growth factor-beta-induced protein ig-h3
P07996	THBS1	Thrombospondin-1
P35442	THBS2	Thrombospondin-2
P35443	THBS4	Thrombospondin-4
Q6ZMP0	THSD4	Thrombospondin type-1 domain-containing protein 4
Q9GZM7	TINAGL1	Tubulointerstitial nephritis antigen-like
P24821	TNC	Tenascin
Q92752	TNR	Tenascin-R
P22105	TNXB	Tenascin-X
Q8WUA8	TSKU	Tsukushi
P13611	VCAN	Versican core protein
P04004	VTN	Vitronectin
Q6PCB0	VWA1	von Willebrand factor A domain-containing protein 1
O00534	VWA5A	von Willebrand factor A domain-containing protein 5A
P04275	VWF	von Willebrand factor

Table S2: Matrisome-associated proteins identified in single-step Azo extracts. 187 of the 324 unique matrisome proteins identified across 3 single-step Azo extraction replicates were matrisome-associated proteins.

UniProt ID	Genes	Protein Name
P01023	A2M	Alpha-2-macroglobulin
O14672	ADAM10	Disintegrin and metalloproteinase domain-containing protein 10
O43184	ADAM12	Disintegrin and metalloproteinase domain-containing protein 12
Q13444	ADAM15	Disintegrin and metalloproteinase domain-containing protein 15
P78536	ADAM17	Disintegrin and metalloproteinase domain-containing protein 17
Q13443	ADAM9	Disintegrin and metalloproteinase domain-containing protein 9
O95450	ADAMTS2	A disintegrin and metalloproteinase with thrombospondin motifs 2
O75173	ADAMTS4	A disintegrin and metalloproteinase with thrombospondin motifs 4
Q6UY14	ADAMTSL4	ADAMTS-like protein 4
P01019	AGT	Angiotensinogen
P02760	AMBP	Protein AMBP
Q9UKU9	ANGPTL2	Angiopoietin-related protein 2
Q9BY76	ANGPTL4	Angiopoietin-related protein 4
P04083	ANXA1	Annexin A1
P50995	ANXA11	Annexin A11
P07355	ANXA2	Annexin A2
P12429	ANXA3	Annexin A3
P09525	ANXA4	Annexin A4
P08758	ANXA5	Annexin A5
P08133	ANXA6	Annexin A6
P20073	ANXA7	Annexin A7
P13497	BMP1	Bone morphogenetic protein 1
P22003	BMP5	Bone morphogenetic protein 5
Q2M2W7	C17orf58	UPF0450 protein C17orf58
P02745	C1QA	Complement C1q subcomponent subunit A
P02746	C1QB	Complement C1q subcomponent subunit B
P02747	C1QC	Complement C1q subcomponent subunit C
Q9BXJ0	C1QTNF5	Complement C1q tumor necrosis factor-related protein 5
P55774	CCL18	C-C motif chemokine 18

P78556	CCL20	C-C motif chemokine 20
Q6YHK3	CD109	CD109 antigen
Q9NNX6	CD209	CD209 antigen
Q05315	CLC	Galectin-10
Q9Y240	CLEC11A	C-type lectin domain family 11 member A
Q86T13	CLEC14A	C-type lectin domain family 14 member A
Q92478	CLEC2B	C-type lectin domain family 2 member B
Q5KU26	COLEC12	Collectin-12
P22792	CPN2	Carboxypeptidase N subunit 2
Q8IUI8	CRLF3	Cytokine receptor-like factor 3
Q6UVK1	CSPG4	Chondroitin sulfate proteoglycan 4
P01034	CST3	Cystatin-C
P04080	CSTB	Cystatin-B
P10619	CTSA	Lysosomal protective protein
P07858	CTSB	Cathepsin B
P53634	CTSC	Dipeptidyl peptidase 1
P07339	CTSD	Cathepsin D
P14091	CTSE	Cathepsin E
P08311	CTSG	Cathepsin G
P09668	CTSH	Pro-cathepsin H
P07711	CTSL	Procathepsin L
P25774	CTSS	Cathepsin S
Q9UBR2	CTSZ	Cathepsin Z
Q14213	EBI3	Interleukin-27 subunit beta
Q9UHF1	EGFL7	Epidermal growth factor-like protein 7
P08246	ELANE	Neutrophil elastase
P00742	F10	Coagulation factor X
P00748	F12	Coagulation factor XII
P00488	F13A1	Coagulation factor XIII A chain
P00734	F2	Prothrombin
P08709	F7	Coagulation factor VII
P00740	F9	Coagulation factor IX
O75063	FAM20B	Glycosaminoglycan xylosylkinase
Q8IXL6	FAM20C	Extracellular serine/threonine protein kinase FAM20C
O00602	FCN1	Ficolin-1
O75636	FCN3	Ficolin-3
P09038	FGF2	Fibroblast growth factor 2
Q5SZK8	FREM2	FRAS1-related extracellular matrix protein 2
Q12841	FSTL1	Follistatin-related protein 1
O95633	FSTL3	Follistatin-related protein 3
Q99988	GDF15	Growth/differentiation factor 15

P35052	GPC1	Glypican-1
O75487	GPC4	Glypican-4
Q9Y625	GPC6	Glypican-6
Q14520	HABP2	Hyaluronan-binding protein 2
P51610	HCFC1	Host cell factor 1
P02790	HPX	Hemopexin
P04196	HRG	Histidine-rich glycoprotein
Q92743	HTRA1	Serine protease HTRA1
P83110	HTRA3	Serine protease HTRA3
Q14005	IL16	Pro-interleukin-16
Q14116	IL18	Interleukin-18
P18510	IL1RN	Interleukin-1 receptor antagonist protein
P19827	ITIH1	Inter-alpha-trypsin inhibitor heavy chain H1
P19823	ITIH2	Inter-alpha-trypsin inhibitor heavy chain H2
Q06033	ITIH3	Inter-alpha-trypsin inhibitor heavy chain H3
Q14624	ITIH4	Inter-alpha-trypsin inhibitor heavy chain H4
Q86UX2	ITIH5	Inter-alpha-trypsin inhibitor heavy chain H5
P21583	KITLG	Kit ligand
P01042	KNG1	Kininogen-1
P09382	LGALS1	Galectin-1
P17931	LGALS3	Galectin-3
P47929	LGALS7	Galectin-7
O00214	LGALS8	Galectin-8
O00182	LGALS9	Galectin-9
P49257	LMAN1	Protein ERGIC-53
P28300	LOX	Protein-lysine 6-oxidase
Q08397	LOXL1	Lysyl oxidase homolog 1
O00187	MASP2	Mannan-binding lectin serine protease 2
P21741	MDK	Midkine
Q9H1U4	MEGF9	Multiple epidermal growth factor-like domains protein 9
P03956	MMP1	Interstitial collagenase
P50281	MMP14	Matrix metalloproteinase-14
Q99542	MMP19	Matrix metalloproteinase-19
P08253	MMP2	72 kDa type IV collagenase
O75900	MMP23B	Matrix metalloproteinase-23
P22894	MMP8	Neutrophil collagenase
P14780	MMP9	Matrix metalloproteinase-9
P15941	MUC1	Mucin-1
P98088	MUC5AC	Mucin-5AC
Q9HC84	MUC5B	Mucin-5B
Q32P28	P3H1	Prolyl 3-hydroxylase 1

Q8IVL5	P3H2	Prolyl 3-hydroxylase 2
Q8IVL6	P3H3	Prolyl 3-hydroxylase 3
P13674	P4HA1	Prolyl 4-hydroxylase subunit alpha-1
O15460	P4HA2	Prolyl 4-hydroxylase subunit alpha-2
Q9NXG6	P4HTM	Transmembrane prolyl 4-hydroxylase
Q92824	PCSK5	Proprotein convertase subtilisin/kexin type 5
Q96FE7	PIK3IP1	Phosphoinositide-3-kinase-interacting protein 1
P00749	PLAU	Urokinase-type plasminogen activator
P00747	PLG	Plasminogen
Q02809	PLOD1	Procollagen-lysine,2-oxoglutarate 5-dioxygenase 1
O00469	PLOD2	Procollagen-lysine,2-oxoglutarate 5-dioxygenase 2
O60568	PLOD3	Multifunctional procollagen lysine hydroxylase and glycosyltransferase LH3
Q6UX71	PLXDC2	Plexin domain-containing protein 2
Q9UIW2	PLXNA1	Plexin-A1
O75051	PLXNA2	Plexin-A2
O15031	PLXNB2	Plexin-B2
O60486	PLXNC1	Plexin-C1
Q9Y4D7	PLXND1	Plexin-D1
P02775	PPBP	Platelet basic protein
P20742	PZP	Pregnancy zone protein
P05451	REG1A	Lithostathine-1-alpha
Q06141	REG3A	Regenerating islet-derived protein 3-alpha
P23297	S100A1	Protein S100-A1
P60903	S100A10	Protein S100-A10
P31949	S100A11	Protein S100-A11
P80511	S100A12	Protein S100-A12
Q99584	S100A13	Protein S100-A13
Q9HCY8	S100A14	Protein S100-A14
Q96FQ6	S100A16	Protein S100-A16
P26447	S100A4	Protein S100-A4
P06703	S100A6	Protein S100-A6
P05109	S100A8	Protein S100-A8
P06702	S100A9	Protein S100-A9
P25815	S100P	Protein S100-P
P18827	SDC1	Syndecan-1
P34741	SDC2	Syndecan-2
P31431	SDC4	Syndecan-4
Q13214	SEMA3B	Semaphorin-3B

Q9H3S1	SEMA4A	Semaphorin-4A
Q9NPR2	SEMA4B	Semaphorin-4B
Q9H3T3	SEMA6B	Semaphorin-6B
P01009	SERPINA1	Alpha-1-antitrypsin
Q9UK55	SERPINA10	Protein Z-dependent protease inhibitor
P01011	SERPINA3	Alpha-1-antichymotrypsin
P29622	SERPINA4	Kallistatin
P05154	SERPINA5	Plasma serine protease inhibitor
P30740	SERPINB1	Leukocyte elastase inhibitor
P48595	SERPINB10	Serpin B10
P29508	SERPINB3	Serpin B3
P35237	SERPINB6	Serpin B6
P50452	SERPINB8	Serpin B8
P50453	SERPINB9	Serpin B9
P01008	SERPINC1	Antithrombin-III
P05546	SERPIND1	Heparin cofactor 2
P05121	SERPINE1	Plasminogen activator inhibitor 1
P07093	SERPINE2	Glia-derived nexin
P36955	SERPINF1	Pigment epithelium-derived factor
P08697	SERPINF2	Alpha-2-antiplasmin
P05155	SERPING1	Plasma protease C1 inhibitor
P50454	SERPINH1	Serpin H1
Q6FHJ7	SFRP4	Secreted frizzled-related protein 4
Q8IWL2	SFTPA1	Pulmonary surfactant-associated protein A1
P07988	SFTPB	Pulmonary surfactant-associated protein B
P11686	SFTPC	Surfactant protein C
P35247	SFTPD	Pulmonary surfactant-associated protein D
P03973	SLPI	Antileukoproteinase
Q9Y5Y6	ST14	Suppressor of tumorigenicity 14 protein
Q07283	TCHH	Trichohyalin
P01137	TGFB1	Transforming growth factor beta-1 proprotein
P21980	TGM2	Protein-glutamine gamma-glutamyltransferase 2
P01033	TIMP1	Metalloproteinase inhibitor 1
P16035	TIMP2	Metalloproteinase inhibitor 2
P50591	TNFSF10	Tumor necrosis factor ligand superfamily member 10
O43508	TNFSF12	Tumor necrosis factor ligand superfamily member 12
P49765	VEGFB	Vascular endothelial growth factor B
Q9Y5W5	WIF1	Wnt inhibitory factor 1
